# Editorial: Applications of nanomaterials for novel thermotherapy

**DOI:** 10.3389/fchem.2025.1656509

**Published:** 2025-08-12

**Authors:** S. Manjura Hoque, Chandan Srivastava, A. K. M. Akhter Hossain, Navid B. Saleh

**Affiliations:** ^1^ Materials Science Division, Atomic Energy Centre Dhaka, Dhaka, Bangladesh; ^2^ Department of Materials Engineering, Indian Institute of Science, Bangalore, India; ^3^ Department of Physics, Bangladesh University of Engineering and Technology, Dhaka, Bangladesh; ^4^ Fariborz Maseeh Department of Civil, Architectural and Environmental Engineering, University of Texas at Austin, Austin, TX, United States

**Keywords:** hyperthermia, cancer, ferrite nanohybrid, cytotoxicity, *in-vitro*

Several studies in the area of “Applications of Nanomaterials for Novel Thermotherapy” provide plausibility to employing heat generated by nanomagnetism as a cancer treatment mediator. Surgical procedures are challenging, particularly when it comes to reaching the deeply embedded tumors in various parts of the body, particularly when brain cancer is present in specific areas. This is in addition to the hazardous side effects of chemotherapy and radiation therapy, which continue to put patients undergoing these treatments at risk.

Zhang et al. developed PEI-Fe_3_O_4_/pYr-ads-8-5HRE-cfosp-IFNG albumin nanospheres for a possible treatment of hepatocellular carcinoma, one of the lethal forms of cancer. The researchers also developed and validated a plasmid, named pY-ads-8-5HRE-cfosp-IFNG, synthesized using double enzyme digestion and gene sequencing methods. The *in-vitro* and therapeutic efficacy experiments indicated that the CCK8 assay yielded a higher rate of cell inhibition in the group receiving combined radiation-gene therapy and magnetic fluid hyperthermia (MFH), compared to the control groups (P < 0.05). Flow cytometry results showed that the combined treatment group had an apoptosis rate of 42.32% and a necrosis rate of 35.73%, both surpassing those of the controls (P < 0.05). In animal studies, the combined treatment exhibited inhibition rates of 66.67% for mass and 72.53% for volume, which are significantly higher than the controls (P < 0.05). Clinical biochemical assessments and histopathological evaluations revealed no abnormalities. The combination of gene therapy with MFH have demonstrated remarkable success in both *in vitro* and *in vivo* studies. This research presents the PEI-Fe_3_O_4_/pYr-ads-8-5HRE-cfosp-IFNG albumin nanosphere system as a promising integrated approach for treating hepatoma, effectively merging immune gene therapy with hyperthermia.

In another study, Islam et al. focused on synthesizing Mg_1−x_Co_x_Fe_2_O_4_ nanoparticles, followed by physicochemical characterizations to tune the magnetic properties of the particles. The primary objective was to assess how the specific loss power and maximum temperature during local magnetic hyperthermia will vary with the change in their physicochemical properties. The study showed that with the increase in the Co^2+^ content, the lattice parameter, X-ray density, ionic radius, hopping length, bond lengths, and both cation-cation and cation-anion distances were increased. Furthermore, spectral analyses with Raman and FTIR spectroscopy indicated that cation distribution also varied with Co^2+^ content and particle size. Magnetic properties measured with a physical property measurement system showed enhancements in saturation magnetization, coercivity, remanent magnetization, and anisotropy constant, corresponding to higher Co^2+^ content and larger particle sizes. Upon exposure to a radio frequency magnetic field, these nanohybrids displayed an increase in both specific loss power and maximum temperature as the particle size initially increased. However, once particle size peaked, these values began to decline. This phenomenon demonstrated that at small size the particles are monodomain that led to limited increase of magnetic anisotropy due to the presence of Co^2+^ which promote both Néel and Brownian relaxation. Conversely, higher particle size led to multidomain structure giving rise to higher anisotropy for which Néel relaxation cannot occur. There exists a critical size threshold in the anisotropic nanoparticles beyond which hyperthermia efficiency degrade significantly.

CaFe_12_O_19_/MnFe_2_O_4_ composites were prepared by Syed et al., where they combined hard (CaFe_12_O_19_) and soft (MnFe_2_O_4_) magnetic phases utilizing a chemical co-precipitation method. Their goal was to incorporate into the hybrid the characteristics of both spinel and hexaferrites based on Mn and Ca. Their selection of such components in the hybrid materials are notable since both Mn and Ca are biocompatible and biodegradable. The physicochemical characteristics were assessed with X-ray diffraction, Fourier transform infrared spectroscopy, a physical properties measurement system, and transmission electron microscopy. A limited coercivity and small magnetization in the exchange coupled CaFe_12_O_19_/MnFe_2_O_4_ composite were observed. Striking results >50 °C maximum temperature and a specific loss power in the range of 50–350 W/g were reported. Cytotoxicity assessments on Vero cell lines indicated a remarkable survival rate of over 95% for the coated composites.

In a comprehensive review, Qiu and Wu discussed that tumor-specific antigens may be derived from a patient’s own tumor cells or created through genetic mutations, which can serve as a cancer controlling strategy. Therefore, they argue that it is crucial to improve the way tumor-specific antigens are presented to immune cells in anti-tumor immunotherapy. Recent advancements in nanoscience have opened up new avenues for tumor immunotherapy. Engineered nanoparticles have gained significant attention for their advantageous and stable qualities. These NPs can efficiently encapsulate chemotherapy drugs and proteins, allowing for targeted delivery within the body. Common carriers include liposomes, polymeric nanoparticles, and metal nanoparticles. Developing smart NP delivery systems can further optimize antigen presentation, thereby potentially enhancing the immune response against tumors. The review summarizes current strategies for sourcing various tumor-specific antigens and examines how these antigens can be effectively delivered to immune cells through intelligent NPs to strengthen anti-tumor immunity. Additionally, we discuss the challenges and opportunities from a clinical translation viewpoint associated with boosting tumor immune responses through smart delivery of cancer controlling substances. The authors argue that this review will stimulate innovative approaches in cancer treatment using intelligent NPs and further advance the research landscape for cancer vaccines.

